# The Influence of UI Design Attributes and Users’ Uncertainty Avoidance on Stickiness of the Young Elderly Toward mHealth Applications

**DOI:** 10.3390/bs15050581

**Published:** 2025-04-25

**Authors:** Zibin Chen, Jaehwan Lee

**Affiliations:** Department of Industrial Design, Hanyang University, ERICA Campus, Ansan 15588, Republic of Korea; czb2021@hanyang.ac.kr

**Keywords:** mHealth application, UI design attributes, uncertainty avoidance, stimulus–organism–response, stickiness, young elderly users

## Abstract

The advantages of mHealth applications have been widely recognized. However, the existing literature rarely explores how user interface (UI) design and individual cultural values influence elderly users’ mHealth application stickiness, particularly among the young elderly. This study examines how two UI design attributes (usability and aesthetics) and individual uncertainty avoidance (from Hofstede’s cultural dimensions) influence elderly users’ stickiness to mHealth applications. The study used PLS-SEM to analyze survey data from 492 elderly people in China. The research results indicate that uncertainty avoidance cultural values are negatively correlated with psychological engagement. The UI design attributes (aesthetic and usability) are positively correlated with psychological engagement, with learnability (usability sub-attributes) having the most significant impact. Furthermore, the study also reveals the serial mediation role of psychological engagement and user internal experiences (satisfaction and attachment). Notably, this study enriches the current literature on user behavior regarding mHealth applications by elucidating the process of user stickiness, incorporating UI design attributes and individual uncertainty avoidance cultural values. These findings offer valuable theoretical and practical insights.

## 1. Introduction

The widespread adoption of Information and Communication Technology (ICT) in the healthcare field has prompted the gradual replacement of traditional devices with smart health monitoring products based on these technologies (Deka et al., 2025; Papa et al., 2020; Tandon et al., 2024). The World Health Organization (WHO) Global Observatory for eHealth (GOE) defines mobile health (mHealth) as “the practice of medical and public health supported by mobile devices, such as mobile phones, patient monitoring devices, personal digital assistants, and other wireless devices” (World Health Organization, 2011, p. 6). As mHealth technologies continue to evolve and become more widely used, understanding the characteristics and needs of their primary users becomes essential. Among these users, elderly individuals represent a particularly important group due to their increasing healthcare needs and the potential benefits they can derive from mHealth interventions (Tajudeen et al., 2022; J. Sun et al., 2016).

The World Health Organization (WHO) defines elderly individuals as those aged 60 and above. However, definitions of elderly may differ in various cultures or countries (Löckenhoff et al., 2009). In developed nations, the age of 65 is generally seen as the threshold for old age. It is noteworthy that China has a relatively lower retirement age compared to developed countries. According to statistics, the retirement age in China is 50 for women and 60 for men (Tu et al., 2022). Therefore, in China, people aged 50–69 are categorized as “young elderly”, those aged 70–79 as “middle-aged elderly”, and those aged 80 and above as “senior-aged elderly” (X. Sun et al., 2024; Wu et al., 1997). A systematic review of digital literacy among elderly individuals revealed that up to 37% of studies defined elderly people as individuals aged 50 and above (Oh et al., 2021). Furthermore, previous research on China’s young elderly has mainly focused on the 50–69 age group (X. Sun et al., 2024; Pan & Dong, 2023). Compared to middle-aged and senior-aged elderly groups, the young elderly tend to have better physical function, more frequent exposure to modern technology, and the ability to independently engage in consuming and learning to use technological products. They represent a key audience in the “smart aging” model and are also the primary elderly users of mHealth.

In the past decade, global enterprises have made substantial investments in the mHealth industry. According to a report by Statista (2025), the global mHealth market size was approximately USD 148 billion in 2023 and is expected to reach USD 258 billion by 2029. The benefits of mHealth are clear; it plays a positive role in significantly enhancing health habits (Free et al., 2013), alleviating health issues (Changizi & Kaveh, 2017), facilitating self-management among chronic disease sufferers (Beratarrechea et al., 2014; H. Kim et al., 2018), extending healthcare to remote locations (Birkmeyer et al., 2021), and enabling real-time doctor–patient communication (Klasnja & Pratt, 2012). Additionally, in the past decade, academic research on mHealth services/applications for the elderly has been rapidly increasing (Tajudeen et al., 2022; J. Sun et al., 2016). The potential exhibited by mHealth has attracted widespread attention in both the academic and industrial sectors. However, despite mHealth’s efficacy in encouraging proactive health behavioral changes (J. P. Higgins, 2016), these applications must be sufficiently appealing (both in performance and design) to ensure continued use by the elderly. In fact, more than half of smartphone applications fall into disuse post-installation or are deleted after just one use (Tarute et al., 2017). This phenomenon underscores a critical need for enhanced user stickiness (Tarute et al., 2017).

Previous studies have shown that the power of any innovation depends on its design, and a good design or experience further satisfies users’ needs and promotes a positive attitude toward innovation (Tandon et al., 2024). Therefore, it is crucial for mHealth applications to offer a comprehensive user experience to the elderly. The ISO 9241-210 standard defines user experience as “the perceptions and responses of the user that result from the use and/or anticipated use of a system, product, or service” (DIS, 2009). In the mHealth context, this means the overall feeling and experience of the user when interacting with an mHealth application, including expectations before use, sensations during use, and reactions after use. Among these, the development and design of the user interface (UI) are key components in constructing this experience. Good UI design not only meets the basic operational needs of users but also establishes an emotional connection with them (Alipour et al., 2021; Kumaran et al., 2023). Additionally, the user’s psychological engagement and internal experience are closely related to their decision to continue using the service (Fang et al., 2017; Cao et al., 2022; Chea & Luo, 2008). Unfortunately, there is little research on how UI affects the psychological engagement and internal experience of young elderly users or how it subsequently influences their stickiness with mHealth applications. Although previous studies have investigated concepts like UI attractiveness/visual appeal/attractiveness to study psychological engagement, engagement, internal states, and behavioral responses (S. Lee et al., 2011; Fang et al., 2017; Rahardja et al., 2023; Peters et al., 2016), these terms and concepts focus only on the aesthetic aspects of UI. However, for a UI, aesthetics is just one of the core factors. Therefore, it is essential to conduct an in-depth exploration of UI design attributes in mHealth. Specifically, the lack of understanding of which UI design attributes and how these attributes trigger user psychological engagement and internal experience hinders the academic and practical understanding of the process of user stickiness formation in mHealth applications.

Additionally, after reviewing the mHealth-related literature, we found that most studies are centered around examining user behavior or intentions using various behavioral intention models or the technology acceptance model (TAM). These prior studies have established a foundation for research on user behavior and intentions in mHealth. However, the main limitation of these models and theories is their failure to consider the potential influence of cultural factors. Previous studies have found that culture is closely linked to user acceptance and behavior (Chi et al., 2023; Al-Okaily et al., 2020; Salcedo & Gupta, 2021). This discovery offers support and justification for the inclusion of cultural factors in our research, facilitating a deeper understanding of how elderly individuals develop stickiness toward mHealth applications. Therefore, in this study, we selected and incorporated uncertainty avoidance from Hofstede’s cultural dimension (Hofstede et al., 2010) to elucidate this process. Compared to other cultural dimensions (individualism, power distance, masculinity, and long-term orientation), we consider “uncertainty avoidance” to be more appropriate as an exogenous variable in this study for the following reasons: (1) For elderly users, adopting any new technology often involves operational complexity and uncertainty in usage. In contrast, other cultural dimensions may have less impact or relevance in this regard. (2) Previous studies have highlighted that privacy risks contribute to hesitation in usage, especially among elderly Chinese users (Pan & Dong, 2023). Compared to other cultural dimensions, “uncertainty avoidance” seems to be a more reasonable explanation for this phenomenon. (3) The elderly often face cognitive and physiological decline, which limits their ability to learn and adapt to new technologies. As a result, uncertainty in technology use tends to be their primary concern. In contrast, other cultural dimensions, such as long-term orientation or masculinity/femininity, focus more on societal orientations or value differences. When discussing older adults’ technology adoption, concerns about technology-related risks are notably more significant. Therefore, “uncertainty avoidance” is more relevant to the real-world context of this study. Based on these considerations, we find it appropriate to use this factor as one of the exogenous variables in this study.

In addition, it is important to clarify that although Hofstede advocated for analyzing cultural dimensions at the national level, this study is conducted at the individual level. Previous research has typically studied the influence of culture at the level of nations or nationalities, assuming that cultural values within the same country exhibit relatively minor differences, while those between different countries are significantly larger (Kirkman et al., 2006; Hofstede et al., 2010). However, some scholars who have engaged in cross-cultural research believe that culture, as an individual value, exhibits significant differences even within a country (Yoon, 2009; Yoo et al., 2011). Since national culture is considered a macro-level phenomenon, it is often inappropriate for explaining individual-level behaviors and reactions. Currently, several studies have expressed similar views, suggesting that cultural models should be built at the individual level to explore user behaviors and responses (Salcedo & Gupta, 2021; Chi et al., 2023; Sharma et al., 2020). As our research focuses on the stickiness of young elderly users toward mHealth applications, this study utilizes the concept of espoused national cultural values. This concept is defined as the degree to which individuals embrace the cultural values of their nation (Srite & Karahanna, 2006). Therefore, the proposed hypotheses are tested at the individual level in the subsequent relevant sections.

Given the importance of UI in mHealth applications and the potential influence of culture on user behavior and acceptance, it is particularly crucial to gain an in-depth understanding of elderly users’ stickiness to mHealth applications from the perspective of UI design attributes and uncertainty avoidance cultural dimension. This is beneficial for the better promotion of mHealth among the elderly population. Considering these reasons, in this study, we seek to find answers to the following questions:

**RQ1:** How significantly do the UI design attributes of mHealth applications influence the stickiness of young elderly users?
**RQ2:** Does the level of uncertainty avoidance influence the stickiness of young elderly users to mHealth applications?
**RQ3:** In mHealth applications, do UI design attributes and uncertainty avoidance indirectly influence the stickiness of young elderly users through psychological engagement and internal experiences?

This study’s contributions are primarily reflected in the following aspects. Firstly, our research may be among the first to provide theoretical insights into how UI design attributes and uncertainty avoidance culture in a mobile environment influence the user stickiness process. Secondly, this study identifies the aesthetic attributes (visual attractiveness and prototypicality) and usability attributes (completeness, memorability, and learnability) of UI design and uncertainty avoidance as key drivers of user stickiness in mHealth applications. Furthermore, by comparing the strength of the path relationships, we can better understand how these factors influence the stickiness of the elderly. Thirdly, most previous studies have used the TAM and its extended variants (i.e., TAM2, TAM3, UTAUT, and UTAUT2) to explain user behavioral intentions toward mHealth (Deng et al., 2018; Alam et al., 2018; Cho, 2016; Leung & Chen, 2019; Duarte & Pinho, 2019). Unlike the previous literature, this study employs the stimulus–organism–response (S-O-R) framework to construct an integrated model to understand the impact of UI design attributes and uncertainty avoidance on the stickiness of elderly users. This also differs from the research by Elsotouhy et al. (2024), who used ISS model components as stimulus factors in the S-O-R model to predict user (non-elderly user) word of mouth, stickiness, and continued usage intentions for health and fitness applications. Fourthly, this study contributes new insights to the literature on elderly behavior, specifically, stickiness toward mHealth. It demonstrates that psychological engagement and user internal experiences act as serial mediation constructs between UI design attributes, uncertainty avoidance, and the stickiness of elderly users. At the end of the article, we offer practical suggestions based on our findings that can be referred to by relevant businesses and providers in the development and design phases of mHealth applications to better win the favor of elderly users.

The subsequent sections of this research are organized as follows: The Section 2 outlines the theoretical framework, research model, and development of the hypotheses. The Section 3 details the research methodology, encompassing sample selection, data collection, and preliminary quality assessments of the data. The Section 4 reveals the findings from the data analysis. The Section 5 engages in a discussion on the theoretical and practical implications, acknowledges limitations, and suggests avenues for future research. The article ends with the conclusion section.

## 2. Theoretical Background and Research Hypotheses

### 2.1. Stimulus–Organism–Response (S-O-R) Theory

The S-O-R theory was initially proposed by Mehrabian and Russell (1974) and is one of the most renowned models in environmental psychology. According to the S-O-R theory (Mehrabian & Russell, 1974), numerous variables can serve as environmental stimuli (S) that drive the internal state of an organism (O), subsequently influencing the user’s behavioral response (R). Previous research has extensively applied the S-O-R framework across various fields to elucidate the behavioral responses of users following external stimuli, including information systems (Parboteeah et al., 2009), e-commerce (Chopdar & Balakrishnan, 2020; J. Lin et al., 2021), hospitality and tourism (Choi & Kandampully, 2019; Chen et al., 2022; Ben Haobin et al., 2021), and retail (Chang et al., 2011; Kühn & Petzer, 2018).

Based on previous research findings, the S-O-R theory is suitable for explaining how cultural or product attributes serve as stimuli affecting user reactions and behavioral responses. For example, L. Li and Kang (2024) combined Hofstede’s cultural theory with the S-O-R model to explore the motivations of adolescents in online entrepreneurship. Additionally, Fang et al. (2017) explored how the design and performance attributes of applications serve as stimuli for psychological engagement and perceived benefits, further revealing the factors influencing user behavioral engagement. Similar to previous research mediums (different types of websites or applications), mHealth applications are also multi-attribute composite software products, where different combinations of attributes might affect users’ internal experiences, subsequently influencing their stickiness. Therefore, the S-O-R theory provides a viable overarching theoretical framework for this study.

Additionally, there are four main reasons for adopting the S-O-R framework in this study. Firstly, previous TAM theories and their extended variants (i.e., TAM2, TAM3, UTAUT, and UTAUT2) may not be suitable for addressing our research questions (RQ). This consideration stems from the exploratory nature of our study. In other words, while both the S-O-R framework and the TAM are theoretical models used to explain human behavior (particularly that of consumers or users), they differ in their theoretical foundations and focal points. The S-O-R model is emotion-driven, focusing on how external stimuli affect behavioral outcomes through internal psychological processes. In contrast, the TAM is functionality-driven, emphasizing the impact of perceived usefulness and perceived ease of use on individuals’ acceptance and usage of technology. Secondly, the S-O-R theory has been widely applied in user behavior research across various types of mobile applications. In other words, the S-O-R theory has been validated in multiple studies for its applicability in understanding complex human behaviors (Elsotouhy et al., 2024; Ali et al., 2021). Thirdly, the unidirectional structural relationships between the components described by the model provide a concise and structured framework for building an integrated model (Kumar et al., 2021). Fourthly, the flexibility and scalability of S-O-R make it applicable in a broader research context. Therefore, the S-O-R theoretical framework is suitable for studying the stickiness of the elderly toward mHealth applications.

### 2.2. Stimuli: UI Design Attributes and Uncertainty Avoidance

The UI design of mHealth applications refers to the quality and effectiveness of the interface, which are crucial for creating an interface that is both aesthetically pleasing and functional, rather than just focusing on aesthetics. Unfortunately, to date, empirical studies on UI design attributes are scarce. Despite this, three studies have proposed classifications for design attributes (regarding UI design). In a systematic review of interfaces for elderly mHealth applications, N. Liu et al. (2021) proposed three sets of UI design improvements based on the needs of the elderly: perceptual capabilities (visual attractiveness), motor coordination issues (interaction), and cognitive and memory decline (usability). In a study on web design attributes, Faisal et al. (2016) divided UI design attributes into aesthetic quality (appeal) and organizational structure and layout (usability). Additionally, Jung and Yim (2017) included simplicity and consistency (usability) in their discussion of UI design attributes and user intentions when using applications. Although the literature on constructing UI design attributes is limited, we can explore potential design attributes from the extensive body of UI design research literature. These previous research findings provide a basis for systematically proposing UI design attributes in this study.

In order to cover all broader topics related to UI, we extracted literature related to this subject from academic databases such as Google Scholar, Taylor & Francis, Springer, Scopus, Emerald, PubMed, and SAGE. We focused primarily on scientific articles, book chapters, conference proceedings, and publications written in English, including papers published from January 2000 to January 2024. Additionally, we used the following keywords to extract relevant publications: user interface, user interface study, user interface design, application user interface, web user interface, user experience, and interaction design. The results show that attractiveness (such as fonts, colors, symbols, icons, and layout), interaction (such as gestures, text input, and button size), and usability (such as learnability, error prevention, simplicity, and consistency) are the main UI design factors discussed in academia.

Based on the content above and related studies, this research conceptualizes two aspects of UI design that may play a critical role in users’ evaluations of an application’s UI design: (1) aesthetic attributes (the extent of the UI design’s aesthetic/visual appeal in applications) and (2) usability attributes (the degree to which the application’s UI design supports users in completing tasks). Furthermore, based on previous research, this study conceptualizes the aesthetic and usability attributes in UI design as multidimensional structures composed of sub-attributes.

The advantages of fonts, colors, graphics, layouts, and prototypicality in enhancing UI aesthetics have been documented in many studies (Bonnardel et al., 2011; Y. Liu & Zhang, 2019; Odushegun, 2023; Zen & Vanderdonckt, 2014; Miniukovich & Figl, 2023). According to the previous literature, visual appeal (i.e., factors such as fonts, colors, graphics, and layouts) and prototypicality are among the factors that influence user experience (Miniukovich & Figl, 2023; Lindgaard, 2007). Thus, visual attractiveness and prototypicality are incorporated as sub-attributes of UI aesthetics in this study.

Good usability in UI design can highlight the unique features of a product and differentiate it from competitors, thereby creating a more favorable impression of the product/service and leading to a positive interaction experience. The Health-ITUEM provides a generic usability evaluation model for mHealth technologies, which is based on the TAM and ISO 9241-11 (Brown et al., 2013). The Health-ITUEM assesses usability through nine criteria: error prevention, completeness, memorability, information needs, flexibility/customizability, learnability, performance speed, competency, and other outcomes. Building on the research by Brown et al. (2013), Tandon et al. (2024) have noted that usability is a multidimensional structure that can be significantly predicted through completeness, learnability, memorability, and customizability. These sub-attributes have shown high reliability, ranging from 90% to 100% (Brown et al., 2013). Therefore, based on previous research findings, we consider completeness, learnability, memorability, and customizability to be the sub-attributes of UI usability in this study.

#### 2.2.1. UI Aesthetic Attributes: Visual Attractiveness and Prototypicality

Visual attractiveness refers to users’ perception of an mHealth UI’s visual and aesthetic aspects, which typically includes the use of color schemes, icons, and graphics. According to E. T. Higgins (2006), the extent of users’ experiences with a system determines their level of engagement. An aesthetically appealing UI design can spark users’ interest and encourage more frequent use of the application (Peters et al., 2016), while complex interfaces and incomplete information may lead elderly users to abandon the application (Brown et al., 2013). Moreover, Tang et al. (2015) emphasized the importance of application design, suggesting that designers should consider the significance of UI design as it determines the functionality of the application. Such attractive designs have often been recognized in previous studies as a powerful tool that can capture users’ attention and facilitate interaction (Xia et al., 2024; Schaffer et al., 2022; Coursaris & Van Osch, 2016; S. Lee et al., 2011). The previous literature indicates that visually attractive UI design influences the experience of users. This can potentially inspire users to invest more time and effort in using the application and develop a strong enthusiasm for it. Therefore, enhancing the visual attractiveness of an application’s UI is expected to increase user psychological engagement. For example, Tian et al. (2021) reported that an attractive user interface can enhance users’ emotional engagement with a mobile travel app. Shin et al. (2024) suggested that the visual aesthetics of a live-streaming commerce interface can significantly enhance users’ consumption experience in a live-streaming commerce setting. Fang et al. (2017) reported that UI attractiveness plays a significant role in stimulating users’ psychological engagement. Therefore, it is reasonable to infer that visually attractive UI design can enhance user psychological engagement. Therefore, we propose the following hypothesis:

**H1:**  *The visual attractiveness of the UI in mHealth applications is positively correlated with elderly users’ psychological engagement.*

Prototypicality describes the extent to which an object represents its category (Miniukovich & Figl, 2023). For example, compared to a penguin, a robin is considered a more prototypical bird because it fits more closely with the general characteristics people associate with birds (Hage & Miller, 1976). Generally, reducing prototypicality can make a product appear more novel/unique (Veryzer & Hutchinson, 1998), which may enhance the user or consumer experience, thereby promoting their engagement, use, or purchase (Celhay & Trinquecoste, 2015). However, when adopting this strategy, it is essential to consider the perceived risk to users. As Celhay and Trinquecoste (2015) pointed out, user preference for atypical product packaging is negatively influenced by their perceived level of risk. Additionally, research by Miniukovich and Figl (2023) found that strong prototypicality positively impacts the perceived credibility of webpages. These studies indicate that users generally prefer UI designs not to deviate too much from what they perceive as the standard model for a specific industry to avoid making the application/webpage appear unreliable or difficult to use. Furthermore, the previous literature suggests that perceived risk is a significant barrier to user psychological and behavioral engagement with mHealth applications (Tandon et al., 2024; Klaver et al., 2021; Deng et al., 2018; Pan & Dong, 2023), implying that there is a high perceived risk associated with current mHealth applications. Based on the above analysis, we can reasonably hypothesize that the more prototypical the mHealth UI, the higher the user’s psychological engagement. Therefore, the following hypothesis can be proposed to test the relationship between the prototypicality of the UI and elderly psychological engagement:

**H2:**  *The prototypicality of the UI in mHealth applications is positively correlated with elderly users’ psychological engagement.*

#### 2.2.2. UI Usability Attributes: Completeness, Memorability, Learnability, and Customizability

In the context of mobile health, the usability of the UI refers to the degree to which it supports users in completing tasks, primarily encompassing completeness, memorability, learnability, and customizability (Appendix A). Unlike traditional health service systems, mHealth relies on the internet and mobile devices to provide health services. However, compared to young people, interacting with applications on small screens becomes challenging for older adults due to physical limitations (Wilson et al., 2021; Elguera Paez & Zapata Del Río, 2019). Peters et al. (2016) noted that if users find the mobile system difficult to understand and experience interruptions during use, their level of emotional engagement will decrease. Additionally, Gu et al. (2009) observed that users would not use the services if they found the applications difficult to operate, regardless of their usefulness. Therefore, to enhance psychological engagement and stickiness among older adults, mHealth applications should ensure robust functionality and good UI usability. An empirical study confirmed that the ease of use of applications has a significant impact on user psychological engagement (Fang et al., 2017). Therefore, we propose the following hypotheses:

**H3:**  *The completeness of the UI in mHealth applications is positively correlated with elderly users’ psychological engagement.*

**H4:**  *The memorability of the UI in mHealth applications is positively correlated with elderly users’ psychological engagement.*

**H5:**  *The learnability of the UI in mHealth applications is positively correlated with elderly users’ psychological engagement.*

**H6:**  *The customizability of the UI in mHealth applications is positively correlated with elderly users’ psychological engagement.*

#### 2.2.3. Uncertainty Avoidance

Uncertainty avoidance refers to an individual’s tolerance for uncertainty and ambiguity in the face of unpredictable changes (Hofstede et al., 2010). Individuals in cultures with high uncertainty avoidance often fear unknown or uncontrollable events, which reduces the likelihood of engaging with them. This ambiguity induces anxiety and stress. As a result, formal organizations or institutions tend to establish clear rules and norms to prevent or reduce these circumstances. These rules and norms aim to make the future as predictable and manageable as possible. However, it should be noted that cultures with high uncertainty avoidance do not discourage taking risks; rather, the risks taken should be known (Salcedo & Gupta, 2021).

We believe that individuals with high uncertainty avoidance cultural values are less likely to engage psychologically with mHealth applications (i.e., vigor, dedication, and absorption). This reluctance stems from their perception of the unknown risks associated with mHealth apps, a concern particularly prevalent among older users in China. As reported by Pan and Dong (2023), elderly individuals do not know where their data ultimately go or what happens after they authorize such applications to access their information. Given the lack of regulation and oversight for mHealth apps, individuals with high uncertainty avoidance are likely to view them as a threat, which, in turn, reduces their psychological engagement. Based on this reasoning, we propose the following hypothesis:

**H7:**  *In mHealth applications, uncertainty avoidance culture is negatively correlated with the psychological engagement of the elderly.*

### 2.3. Organism: Psychological Engagement and Internal Experience

The concept of engagement has garnered considerable attention across multiple fields. Although many researchers have confirmed the various benefits of user engagement (Cheung et al., 2015; Y. H. Kim et al., 2013; McLean, 2018), there seems to be a significant amount of debate and disagreement regarding the definition, dimensions, and operational aspects of “engagement” (Jacques, 1995; O’Brien & Toms, 2008). Therefore, this study adopts the perspective of Fang et al. (2017) when using the term “engagement”. According to Fang et al. (2017), user engagement can be categorized into psychological engagement, which focuses on the mental state of the user, and behavioral engagement, which focuses on observable actions. Psychological engagement is defined as “the extent of a user’s positive, fulfilling, and app-related psychological state, marked by vigour, dedication, and absorption” (Fang et al., 2017).

Bruce (1998) defined satisfaction as a psychological state that integrates users’ emotional and actual responses to a specific activity. Furthermore, according to Oliver (1999), satisfaction refers to whether the performance and purpose of a product or service meet the user’s expectations. As users interact with an application, they become psychologically engaged once their issues are resolved (Fang et al., 2017). From a psychological perspective, previous studies have shown that flow is a form of highly immersive engagement (Calvo-Porral et al., 2017). A high-psychological-engagement experience usually means that users are satisfied with the experience provided by the application and are willing to invest time and effort in interaction. As noted by Loureiro et al. (2017), users who interact online with brands are more likely to feel satisfied. Additionally, Masrek et al. (2018) observed, in the context of the web and digital libraries, that users’ felt involvement significantly affects their satisfaction. Therefore, we can propose the following hypothesis:

**H8:**  *In mHealth applications, the psychological engagement of elderly users is positively correlated with their satisfaction.*

Unlike satisfaction, attachment is considered a higher-order concept (Y. Li et al., 2021). According to the attachment theory proposed by Bowlby and Bowlby (2012), an attachment relationship develops when an individual associates with a specific object. Product/brand attachment has been a focal point of interest (Pedeliento et al., 2016; Wei et al., 2022; Guru et al., 2024). As a psychological link between users and services, product/brand attachment can have a lasting and stable impact on user behavior (Carroll & Ahuvia, 2006). The formation of attachment to technology or services occurs as follows: initial interactions with the technology or service trigger positive emotions, which gradually evolve into a deep attachment through continued interaction (Thomson et al., 2005). In other words, the formation of attachment to products/brands requires time and high user psychological engagement. The functionalities offered by such products/services provide users with continuous and convenient support, which, in turn, makes them more likely to develop positive emotions toward mHealth (J. Li et al., 2020). Additionally, previous studies have provided support for the relationship between users’ psychological engagement and attachment. Thomson et al. (2005) noted that the frequency and depth of user interactions with a brand can enhance emotional attachment. Y. Li et al. (2021) similarly observed, in live-streaming platforms, that increased interaction and identification enhanced users’ emotional attachment to their hosts. X. F. Zhang et al. (2021) also confirmed this association, finding that patients’ deep interactions and personalized feedback with products contribute to emotional attachment to mHealth. Based on these reasons, the following hypothesis can be proposed to explain the relationship between the psychological engagement of elderly users and emotional attachment in mHealth applications:

**H9:**  *In mHealth applications, the psychological engagement of elderly users is positively correlated with their attachment.*

### 2.4. Response: Stickiness

Zott et al. (2000) defined stickiness as a business’s ability to attract and retain users. Marchand and Davenport (2000) noted that user stickiness implies that a product/service can provide users with a positive experience, thereby encouraging their continued use. In other words, even if other companies engage in marketing campaigns on social networks, the deep loyalty of customers to a specific company’s social network (manifested as strong stickiness) ensures they will continue to revisit and use that company’s social network (D. Li et al., 2006). J. C. C. Lin (2007) understood this user stickiness as the potential, unconscious behavior of users returning to that company’s social network. Thus, platforms with higher stickiness may gain more traffic and user visit duration, thereby promoting users’ willingness to engage in platform activities. To summarize, the academic definition of stickiness encompasses two key elements: visit duration and user retention (M. Zhang et al., 2017). Based on this, we adopted the definition of stickiness provided by M. Zhang et al. (2017), which includes both the visit duration and user retention.

Preliminary research indicates that user satisfaction directly impacts user stickiness. According to Maqableh et al. (2021), user stickiness on the Facebook social platform is significantly influenced by satisfaction. Shen et al. (2022) and Elsotouhy et al. (2024) found the same relationship in live-streaming commerce platforms and fitness applications, respectively. Shao et al. (2020) demonstrated that satisfaction positively promotes user stickiness to SNS. Wang et al. (2016) noted that satisfaction is a key determinant of users’ stickiness on group buying websites. Furthermore, in terms of attachment, past research has shown that emotional attachment increases user stickiness to products/brands (Y. Li et al., 2021; Gao et al., 2022; Madina & Kim, 2021). These prior studies provide support for our understanding of the relationships between satisfaction, attachment, and stickiness. Based on these findings, the following hypotheses can be reasonably proposed:

**H10:**  *In mHealth applications, the satisfaction of elderly users is positively correlated with their stickiness.*

**H11:**  *In mHealth applications, the attachment of elderly users is positively correlated with their stickiness.*

**Figure 1:**
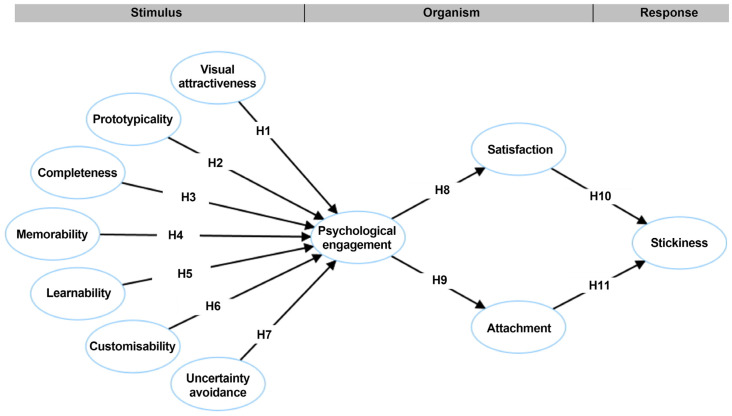
The research model.

The research model is shown in Figure 1.

## 3. Methods

### 3.1. Sampling and Data Collection

A large sample size significantly enhances the generalizability and reliability of results. Therefore, to improve the reliability of our outcomes, we collected samples from 597 elderly Chinese individuals through the Wenjuanxing (a platform providing functions equivalent to Amazon Mechanical Turk) online platform in September 2024. In this research, we used a “self-selection” sampling method, which allows participants to join the survey voluntarily. The dual constraints imposed certain limitations on available resources. Under these circumstances, self-selection sampling enabled the efficient gathering of a large number of responses, especially through online or social media distribution channels. As Rizzo et al. (2004) suggest, this is the most suitable strategy when conducting digital surveys. To protect respondents’ personal privacy, it was clearly stated at the beginning of the questionnaire that the data would only be used for academic research purposes, and no personal information was required. Additionally, the first page of the survey includes an “Informed Consent Statement” section, where participants must select the option “I have fully understood and agree to participate in this study” before completing the questionnaire to ensure they have their right to be informed. After submitting the questionnaire, we asked them to recommend colleagues, relatives, or friends with experience using mHealth applications to fill out the survey to expand the sample size.

### 3.2. Measurement

All measurement items for the variables in the study were sourced from previous research. Appendix B displays the scale items for each construct. For example, visual attractiveness was assessed through measurement items such as “*The user interface design of mobile health apps looks clean*”, “*The user interface design of mobile health apps is fascinating*”, *and* “*The user interface design of mobile health apps is sophisticated*”. These measurement items were scored using a Likert seven-point scale, ranging from 1 = “strongly disagree” to 7 = “strongly agree”. Additionally, we meticulously revised the measurement items to enhance their alignment with the objectives of this study. The questionnaire was initially written in English and then translated into the respondents’ native language (Chinese). This part of the work was performed by a scholar proficient in both English and Chinese to ensure that the translated measurement items were consistent with the original content. Before officially distributing the questionnaire, a pilot test was conducted with four Chinese elderly individuals (aged 52, 56, 61, and 65), who were asked to complete the questionnaire continuously in an undisturbed setting. This was to ensure the clarity and simplicity of the questions and the overall duration of the responses. The results showed that the participants in the pilot test completed their responses continuously, with their response times ranging from 448 s to 1161 s. Furthermore, following their recommendations, we subtly adjusted the phrasing of certain questions to elevate the overall clarity and understandability of the questionnaire.

### 3.3. Preliminary Quality and Common Method Bias Checks

Before analysis, we conducted two preliminary checks on the raw data to ensure data quality. First, based on the pilot test with four elderly participants, we excluded questionnaires with response times significantly below 448 s, such as around 120 s. Second, we checked for missing values and found none. Ultimately, 492 questionnaires were used for subsequent analysis. According to Westland (2010)’s statistical techniques for the minimal sample size required for structural equation modeling, the sample size of this study meets the expected requirements. The detailed demographic information of the respondents is shown in Table 1.

To verify the reliability of the data, we checked for the potential impact of common method bias (CMB). According to Birkmeyer et al. (2021), this type of bias is not related to the data themselves but primarily associated with the survey method used. Additionally, Podsakoff and Organ (1986) pointed out that when the indicators of exogenous and endogenous structures are evaluated by the same source or individual, the source’s bias may affect both indicators in the same manner, leading to distortion. Therefore, to assess the impact of CMB, we conducted Harman’s one-factor test (Harman, 1976). Typically, in exploratory factor analysis, the absence of this bias is indicated only if multiple factors rather than a single factor are extracted, and the variance of the first factor is less than 50%. In this study, 11 factors with eigenvalues greater than 1 were extracted, accounting for a cumulative variance of 81.702%, and the variance of the first extracted factor was 32.871%, which is below the recommended threshold. These results suggest that there is no common method bias in our study. Additionally, we also tested for multicollinearity. The results showed that all variance inflation factors (VIFs) were between 2.022 and 4.438, below the recommended threshold of 5, indicating that there is no collinearity in the data.

## 4. Data Analysis and Results

We utilized descriptive statistics and Partial Least Squares Structural Equation Modeling (PLS-SEM) using the SmartPLS 4 software for data analysis. This study opted for PLS-SEM primarily due to the following considerations. Firstly, as an exploratory study, PLS-SEM is more suitable for exploring and predicting relationships between structures than covariance-based SEM (CB-SEM) (Chin, 1998a), thus aiding in addressing the research questions (RQs) posed by this study. Specifically, this study focuses on theoretical construction (particularly regarding elderly users’ stickiness to mobile applications) and hypothesis exploration rather than strict testing based on existing theory. Secondly, it does not have stringent requirements regarding data distribution (Hair et al., 2019). Thirdly, PLS-SEM has advantages in estimating and assessing mediating effects. Fourthly, PLS-SEM offers greater flexibility in handling multiple latent variables, numerous measurement indicators, and complex models. Compared to CB-SEM, it is less prone to issues such as model identification errors. Given these points, we believe that PLS-SEM is an appropriate data analysis technique for this study.

### 4.1. Measurement Model Assessment

This study assessed the measurement model through convergent validity, discriminant validity, and construct reliability. As shown in Table 2, all factor loadings were greater than 0.7, surpassing the threshold of 0.7 recommended by Fornell and Larcker (1981). The variables’ Cronbach’s alpha and construct reliability values all met the standard of being greater than 0.7 (Anderson & Gerbing, 1988). All constructs’ average variance extracted (AVE) values exceeded the recommended threshold of 0.5, indicating the strong convergent validity of the measurement model.

We tested discriminant validity using the Fornell–Larcker Criterion (Hair et al., 2019), and Table 3 shows that the square root of the AVE (average variance extracted) for each construct (values on the diagonal) is higher than its correlations with any other construct. Additionally, following the recommendation of Henseler et al. (2015), we further corroborated the discriminant validity through the heterotrait–monotrait ratio (HTMT). Table 4 indicates that all HTMT thresholds are below 0.85, confirming the discriminant validity of all variables. Overall, the reliability of the measurement in this study meets all the criteria previously proposed in research, and the model’s reliability is confirmed.

### 4.2. Structural Model Assessment

R-square ($R^{2}$), path values, and their respective t-values were determined using a bootstrapping technique involving 5000 samples. The cross-validated redundancy index ($Q^{2}$) was calculated using the blindfolding algorithm with an omission distance value of 7.

#### 4.2.1. Model Fit

**Figure 2:**
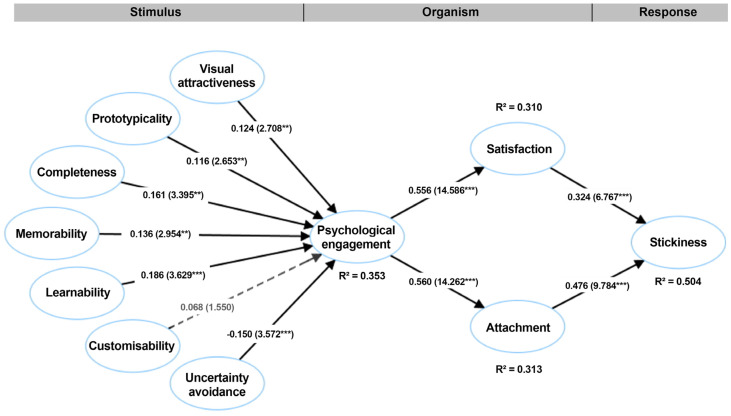
Results of the PLS-SEM model ($Q^{2}$, $R^{2}$, t, and *β*). Significance level: *** *p* < 0.001, ** *p* < 0.01.

Chin (1998b) proposed that the predictive relevance of a structural model could be assessed by calculating the $Q^{2}$ values for endogenous variables. A $Q^{2}$ value greater than 0 indicates that the model has predictive relevance for endogenous variables. In this study, the $Q^{2}$ for psychological engagement is 0.296, for satisfaction it is 0.261, for attachment it is 0.257, and for stickiness it is 0.437, indicating that the model has good predictive power. The explanatory power of the model can be assessed through $R^{2}$ values, which represent the proportion of variance explained by exogenous variables. As shown in Figure 2, the $R^{2}$ for psychological engagement is 0.353, for satisfaction it is 0.310, for attachment it is 0.313, and for stickiness it is 0.504, demonstrating that the model has good explanatory power. Additionally, goodness of fit (GOF) is a criterion for measuring model fit, and a GOF value exceeding 0.36 indicates a high degree of fit (Tenenhaus et al., 2005). The GOF value of this study is 0.550, meeting the criteria for a high degree of fit. Lastly, according to Tenenhaus et al. (2005), a Standardized Root Mean Square Residual (SRMR) value less than 0.080 indicates a good model fit. In this study, the SRMR value is 0.032, satisfying this criterion. Overall, our research model demonstrates a high degree of fit.

#### 4.2.2. Hypothesis Test Results

After assessing the model’s fit, we conducted path analysis to determine whether the relationships between the construction of the structural model were statistically significant. The beta (*β*) values reflect the strength of the relationships between variables, while the t-values indicate their statistical significance. If the path coefficients are statistically significant (i.e., t > 1.96), the relationships between these variables can be considered valid. As shown in Table 5, standardized path coefficients for uncertainty avoidance (*β* = −0.150; t = 3.572) and UI design attributes, namely, visual attractiveness (*β* = 0.124; t = 2.708), prototypicality (*β* = 0.116; t = 2.653), completeness (*β* = 0.161; t = 3.395), memorability (*β* = 0.136; t = 2.954), and learnability (*β* = 0.186; t = 3.629), demonstrated statistically significant positive effects on the psychological engagement of elderly users with mHealth applications. Therefore, H1–H5 and H7 were supported. This indicates that the UI design of mHealth promotes users’ psychological engagement, while individual-level espoused national cultural values (uncertainty avoidance) inhibit their psychological engagement. However, the impact of customizability (*β* = 0.068; t = 1.550) on psychological engagement in mHealth applications is negligible; therefore, H6 is rejected. This indicates that elderly users in China do not place a high level of attention on the personalization of mHealth UI.

The standardized path coefficients for psychological engagement (*β* = 0.556 and t = 14.586; *β* = 0.560 and t = 14.262) showed statistically significant positive effects on the satisfaction and attachment of elderly users with mHealth applications, with *p* < 0.001. Therefore, H8 and H9 were supported. This established path indicates that psychological engagement can effectively explain users’ satisfaction with and attachment to mHealth. As predicted, the standardized path coefficients for internal user experiences—satisfaction (*β* = 0.324; t = 6.767) and attachment (*β* = 0.476; t = 9.784)—demonstrated statistically significant positive effects on increasing the stickiness of elderly individuals using these applications, with *p* < 0.001. Thus, H10 and H11 were also supported. This suggests that users’ stickiness to mHealth can be explained by satisfaction and attachment, with attachment having a stronger explanatory power than satisfaction.

To gain a deeper understanding of the mediating effects of psychological engagement and internal experiences (satisfaction and attachment) on the stickiness of elderly users toward mHealth applications, we conducted a mediation effect analysis. The results of the mediation effects are shown in Table 6. The findings indicate that customizability was the only factor that failed to exert a significant influence on user stickiness via (1) psychological engagement and satisfaction (β = 0.012; t = 1.450) and (2) psychological engagement and attachment (β = 0.018; t = 1.502). Furthermore, uncertainty avoidance and most UI design attributes have significant indirect effects on the stickiness of elderly users toward mHealth applications. Specifically, these factors primarily influence elderly users’ stickiness through two mediation pathways: (1) psychological engagement and satisfaction and (2) psychological engagement and attachment. The results of the mediation effect analysis underscore the serial mediating roles of psychological engagement and internal user experiences (satisfaction and attachment).

## 5. Discussion

The importance of user stickiness in promoting commercial success and sustaining relationships between users and products/services has been widely acknowledged in the scientific community (J. C. C. Lin, 2007; Hsu & Lin, 2016; Wang et al., 2016; S. Kim et al., 2016). Exploring the process of producing user stickiness has become a significant topic in both academic and practical fields. To date, although numerous studies have examined users’ stickiness toward mHealth (Yin et al., 2021; Elsotouhy et al., 2024; Soni et al., 2021; Guo et al., 2020), few have explored the stickiness of elderly user groups from the perspective of UI design attributes and cultural factors. This study is unique, as it explores the UI design factors and uncertainty avoidance culture affecting elderly people’s stickiness toward mHealth applications based on the S-O-R theory. Specifically, we investigated two aspects of mHealth application UI design: aesthetic attributes (visual attractiveness and prototypicality) and usability attributes (completeness, memorability, learnability, and customizability). We explored how these sub-attributes, along with uncertainty avoidance, drive elderly users’ stickiness through psychological engagement and internal user experiences. This empirical research has revealed some significant findings that were not previously covered in previous studies.

Firstly, this study found that UI design plays an important role in the stickiness of mHealth applications among elderly users. The results confirmed that visual attractiveness and prototypicality (UI aesthetic attributes), completeness, memorability, and learnability (UI usability attributes) are UI drivers of elderly users’ stickiness toward mHealth applications. Notably, visual attractiveness and learnability played the most significant roles within their respective categories of aesthetics and usability, illustrating the varied impacts of different UI sub-attributes on driving elderly users’ stickiness. These UI design attributes were positively correlated with elderly users’ psychological engagement, further supporting the findings of previous research (Fang et al., 2017). Furthermore, enhancing user psychological engagement increased user satisfaction and attachment levels, thereby triggering stickiness. The comprehensive results of this study indicate that user psychological engagement is a crucial predictor of satisfaction and attachment, which, in turn, are significant predictors of user stickiness. This conclusion aligns with previous research findings (Elsotouhy et al., 2024; Ali et al., 2021; C. H. Lee et al., 2018; Gao et al., 2022; Lien et al., 2017).

Secondly, the findings reveal that compared to the aesthetic attributes of UI, the usability attributes have a more positive impact on the psychological engagement of elderly users. This result aligns with Tandon et al. (2024), confirming the importance of UI usability for elderly engagement in mHealth applications. However, in terms of UI usability attributes, we observed that elderly users place greater importance on the learnability of the mHealth application’s UI, which appears to differ from the observations of Tandon et al. (2024). They noted that customizability more significantly influenced elderly users’ perceptions of mHealth usability (Tandon et al., 2024). However, in our study, customizability did not have a positive effect on users’ psychological engagement. The reasons for these differences may vary, but we believe three main reasons could be the most significant. Firstly, this study primarily focuses on the stickiness of young elderly Chinese users toward mHealth applications, whereas Tandon et al. (2024) mainly targeted elderly people in various states of Northern India. We consider the differences between populations (manifested as cultural and national distinctions) to be the primary cause of this discrepancy in usability perceptions, which has been widely discussed and documented in the prior literature (Wallace et al., 2013; Clemmensen et al., 2007; Alexander et al., 2021). Secondly, significant differences in the educational backgrounds of the study subjects could be a secondary reason for these discrepancies. Thirdly, this difference may be due to the fact that elderly users in China prioritize the practicality and user-friendliness of mHealth UI while having a relatively low demand for personalization. Although there are differing conclusions regarding usability specifics, our study underscores the importance of mHealth application usability for elderly users’ behavior (stickiness in this study).

Thirdly, the study also found a negative correlation between uncertainty avoidance culture and psychological engagement. Prior research has already linked cultures with high uncertainty avoidance to individuals’ risk tolerance and avoidance tendencies (Yoo et al., 2011; Salcedo & Gupta, 2021). When it comes to adopting new technology, particularly when the technology is not regulated by official institutions or government agencies, users with high uncertainty avoidance cultural values may feel anxiety and stress. This often leads them to reject such technologies. We found that the significant statistical results for H7 not only support the negative relationship between the two but also align with the literature on the relationship between uncertainty avoidance culture and technology adoption (Hoehle et al., 2015; Salcedo & Gupta, 2021; Al-Okaily et al., 2020).

Fourth, this study revealed that psychological engagement and user internal experiences (satisfaction and attachment) serve as serial mediators between uncertainty avoidance, UI design attributes, and the stickiness of elderly users. The results indicate that uncertainty avoidance and most UI design attributes have a significant indirect effect on elderly users’ stickiness toward mHealth applications. Specifically, psychological engagement, satisfaction, and attachment mediate the influence of these two factors (UI design attributes and uncertainty avoidance) on elderly users’ stickiness. Of these, the serial mediating effects of psychological engagement and attachment were more pronounced than those of psychological engagement and satisfaction.

### 5.1. Theoretical Implications

This study contributes to several theoretical respects. Firstly, we developed an integrated model through the S-O-R framework that delineates the impact of uncertainty avoidance and UI design attributes on the user stickiness of mHealth applications. This model theoretically unveils the relationships between uncertainty avoidance, UI design attributes, psychological engagement, internal user experiences, and stickiness.

Secondly, this research adds new insights into the behavior (in this study, stickiness) of elderly users toward mHealth applications by exploring the factors constituting UI design attributes. Although previous research has delved into the influence of UI attractiveness on user behavior (Peters et al., 2016; Fang et al., 2017; Rahardja et al., 2023), there has been a lack of systematic examination of other UI design attributes that may influence user behavior. Our research makes a contribution to bridging this gap. This study demonstrates that not all UI design sub-attributes stimulate young elderly users’ psychological engagement to the same extent, which, in turn, drives their stickiness. Specifically, the PLS-SEM results indicate that visual attractiveness (UI aesthetic attribute) and learnability (UI usability attribute) are critical UI design sub-attributes that stimulate young elderly users’ psychological engagement. These factors collectively drive the stickiness of young elderly users.

Thirdly, this study enriches this body of knowledge by incorporating uncertainty avoidance from Hofstede’s cultural dimensions. In mHealth-related research, although there has been much study on user adoption behavior and intentions, the influence of culture has not been considered previously. Our study highlights the role of the cultural value of uncertainty avoidance in user stickiness. The study shows that the higher the cultural values of uncertainty avoidance, the lower the individual’s stickiness to mHealth applications. It demonstrates the role of an individual concept of espoused national cultural values in either facilitating or impeding user stickiness to mHealth applications. Furthermore, many previous studies have treated culture as a monolithic construct and compared it between specific countries, rather than focusing on the cultural values held at the individual level. This study enriches the development of related theories by focusing on the cultural values embraced at the individual level.

### 5.2. Practical Implications

This study reveals several significant findings with practical implications. First, our research suggests that developers and designers of mHealth applications should focus on two categories of UI design attributes—namely, aesthetic and usability attributes—to enhance the psychological engagement and internal experiences of elderly users, as these attributes significantly influence their stickiness. Specifically, in terms of UI aesthetic attributes, UIs that are visually attractive and prototypical can enhance elderly users’ psychological engagement. In fact, the visual attractiveness and prototypicality of a UI do not conflict; previous studies indicate that a typical UI can reduce perceived risks for elderly users and significantly increase their degree of acceptance. This can be understood as follows: designers should enhance visual attractiveness without excessively deviating from the industry standards of UI prototypicality. Regarding UI usability attributes, these should be a priority for designers and developers, such as learnability, completeness, and memorability. An intuitive design approach plays a crucial role in enhancing the usability of UI for elderly users (Tandon et al., 2024), making it easier for them to discover and use features and services within mHealth applications. We believe this intuitive design approach is mainly reflected in the following aspects:

(1) Progressive disclosure: We recommend that developers of mHealth applications do not display all functionalities at once but gradually introduce more features based on the progress of elderly users to avoid information overload, leading to lower learnability and memorability.
(2) Consistency within the interfaces of mHealth applications should be maintained, including the use of colors, fonts, layouts, and interactive elements, which helps elderly users learn and adapt to the application more quickly.

Secondly, in terms of uncertainty avoidance culture, enterprises related to mHealth applications might consider using behavioral data-mining techniques to deeply understand user cultures in different regions, such as user personas. This technique/method can effectively segment and cluster similar users. This enables relevant enterprises to develop precise UI design strategies for that segment or region, enhancing user psychological engagement and internal experiences and thus increasing user stickiness. Therefore, to enhance psychological engagement, internal experiences, and stickiness among elderly users, mHealth applications need to comprehensively consider the impact of UI design and culture.

Thirdly, our study shows that attachment has a stronger influence on the stickiness of elderly users of mHealth applications compared to satisfaction. This finding suggests that, in addition to focusing on the usability of the UI, related enterprises should also pay attention to the level of user attachment. Therefore, we recommend that practitioners, after fulfilling users’ interactive needs with the interface, should also consider the quality of information provided by mHealth applications. This may help to enhance users’ perceived usefulness, thereby increasing their level of attachment. This is primarily because the explanatory power of attachment in this study is 31.3%, indicating that an additional 68.7% of the variance is explained by factors outside of the study model.

### 5.3. Limitations and Future Research Directions

This study’s limitations provide directions for future research. First, the sample consisted solely of elderly Chinese individuals. Therefore, we suggest that future work could validate the research model across different regions or countries to enhance the applicability of the conclusions. Second, the sampling method used in this study (self-selected sampling) is a non-probability sampling technique. As a result, the collected data may not be representative of all users, which could affect the generalizability of the findings. Future research should adopt probability sampling methods and extend the sample to include non-users in order to explore the differences between users and non-users. Third, this study utilized the S-O-R framework to explore factors that might stimulate user psychological engagement and internal experiences and influence stickiness from the perspective of design and cultural factors. However, other performance- and functionality-related variables, such as stability, response time, ease of use, and usefulness, may also play significant roles in influencing user psychological engagement, internal experiences, and stickiness. Therefore, future research could utilize various determinants of UI design attributes and cultural factors to improve the model of study. Fourth, our research model did not consider the impact of moderating variables, such as demographic variables and personal innovativeness variables. Further research could integrate these moderating variables into the model and assess their effects, which could be an interesting avenue to explore.

## 6. Conclusions

This study significantly advances our theoretical understanding by developing an integrated S-O-R model to clarify how uncertainty avoidance and UI design attributes influence elderly users’ stickiness to mHealth applications. We address existing research gaps by systematically analyzing UI design sub-attributes, revealing that visual attractiveness and learnability are particularly critical in stimulating psychological engagement among young elderly users. Furthermore, by incorporating Hofstede’s cultural dimension of uncertainty avoidance, this research highlights how cultural values at an individual level significantly influence user stickiness, thus enriching the literature on user behavior in mHealth applications.

From a practical perspective, this research emphasizes that mHealth developers should prioritize aesthetic and usability attributes in UI design, enhancing visual attractiveness without compromising UI prototypicality. Employing intuitive design strategies such as progressive disclosure and consistent interface elements can significantly enhance elderly users’ psychological engagement and stickiness. Additionally, enterprises should leverage behavioral data-mining techniques to create culturally tailored UI designs, further enhancing user psychological engagement and stickiness.

Lastly, this study acknowledges its limitations, such as the sample’s limited geographic and demographic scope, the use of non-probability sampling methods, and the omission of potentially influential moderating variables. Future research should thus aim for broader geographic validation, employ probability sampling, and integrate additional performance-related factors and moderating variables to further refine the theoretical model and its practical applicability.

## Figures and Tables

**Table 1:** Demographic profile (*n* = 492).

Socio-Demographic Variable	Frequency	Percentage (%)
Age		
50–54	210	42.7
55–60	161	32.7
61–65	112	22.8
66–69	9	1.8
Gender		
Male	231	47.0
Female	261	53.0
Education qualification		
Primary school diploma	75	15.2
Middle school diploma	235	47.8
High school diploma	97	19.7
Bachelor’s degree	47	9.6
Master’s degree	25	5.1
Doctorate degree	13	2.6
Application usage		
Less than 1 year	186	37.8
1–3 year	235	47.8
More than 3 years	71	14.4
Preferred features		
Health monitoring (e.g., body temperature, blood pressure, blood glucose, and heartbeat)	104	21.1
For emergency (e.g., calling for help automatically, providing vital medical information in an emergency like allergies and medical conditions)	77	15.7
Self-assessment or self-diagnose (e.g., checking health status with apps by yourself)	26	5.3
Finding suitable doctors and hospitals and making an appointment	44	8.9
Knowledge about health and health preservation information	33	6.7
Helping with healthy diet (e.g., healthy recipes, calories calculator, and food diary)	56	11.4
Fitness and exercises (step counter and exercise guide)	75	15.2
Communicating with a doctor online	27	5.5
Communicating with people who have the same health issue	50	10.2

**Table 2:** Descriptive statistics, factor loading coefficients, and convergent validity.

Construct	Indicators	Factor Loading	Cronbach’s Alpha	CR	AVE
Visual attractiveness (VA)	VA1	0.821	0.916	0.924	0.706
	VA2	0.852			
	VA3	0.801			
	VA4	0.785			
	VA5	0.885			
	VA6	0.892			
Prototypicality (PT)	PT1	0.866	0.887	0.910	0.814
	PT2	0.917			
	PT3	0.922			
Completeness (COM)	COM1	0.902	0.889	0.908	0.768
	COM2	0.877			
	COM3	0.820			
	COM4	0.904			
Memorability (MEM)	MEM1	0.923	0.919	0.920	0.861
	MEM2	0.929			
	MEM3	0.931			
Learnability (LRN)	LRN1	0.904	0.930	0.931	0.827
	LRN2	0.917			
	LRN3	0.898			
	LRN4	0.919			
Customizability (CTM)	CTM1	0.892	0.923	0.929	0.812
	CTM2	0.896			
	CTM3	0.903			
	CTM4	0.913			
Uncertainty avoidance (UA)	UA1	0.887	0.919	0.919	0.804
	UA2	0.888			
	UA3	0.900			
	UA4	0.912			
Psychological engagement (PEN)	PEN1	0.919	0.919	0.920	0.860
	PEN2	0.937			
	PEN3	0.926			
Satisfaction (SAT)	SAT1	0.924	0.941	0.942	0.849
	SAT2	0.915			
	SAT3	0.917			
	SAT4	0.929			
Attachment (ATT)	ATT1	0.914	0.959	0.960	0.831
	ATT2	0.908			
	ATT3	0.911			
	ATT4	0.917			
	ATT5	0.909			
	ATT6	0.909			
Stickiness (ST)	ST1	0.934	0.928	0.929	0.875
	ST2	0.935			
	ST3	0.936			

**Table 3:** Discriminant validity of AVE square roots.

	ATT	COM	CTM	LRN	MEM	PT	PEN	SAT	ST	UA	VA
ATT	0.912										
COM	0.344	0.876									
CTM	0.273	0.203	0.901								
LRN	0.422	0.322	0.221	0.909							
MEM	0.348	0.336	0.247	0.435	0.928						
PT	0.321	0.323	0.225	0.269	0.247	0.902					
PEN	0.560	0.384	0.262	0.443	0.389	0.334	0.927				
SAT	0.558	0.255	0.223	0.351	0.279	0.359	0.556	0.921			
ST	0.657	0.298	0.250	0.388	0.320	0.326	0.550	0.590	0.935		
UA	−0.321	−0.260	−0.227	−0.418	−0.265	−0.234	−0.384	−0.286	−0.293	0.897	
VA	0.272	0.216	0.21	0.294	0.261	0.254	0.335	0.216	0.254	−0.283	0.840

**Table 4:** Heterotrait–monotrait ratio.

	ATT	COM	CTM	LRN	MEM	PT	PEN	SAT	ST	UA	VA
ATT											
COM	0.364										
CTM	0.289	0.221									
LRN	0.446	0.352	0.237								
MEM	0.370	0.366	0.265	0.471							
PT	0.342	0.359	0.245	0.291	0.270						
PEN	0.596	0.419	0.283	0.478	0.423	0.364					
SAT	0.586	0.276	0.238	0.375	0.299	0.389	0.597				
ST	0.696	0.321	0.269	0.417	0.345	0.352	0.595	0.630			
UA	0.342	0.286	0.245	0.453	0.288	0.257	0.418	0.308	0.318		
VA	0.287	0.237	0.222	0.318	0.284	0.283	0.361	0.230	0.274	0.308	

**Table 5:** Estimates of the model’s structural path coefficients.

Hypotheses		Beta	t	*p*	Conclusion
H1	Visual attractiveness → psychological engagement	0.124	2.708	0.007 **	Supported
H2	Prototypicality → psychological engagement	0.116	2.653	0.008 **	Supported
H3	Completeness → psychological engagement	0.161	3.395	0.001 **	Supported
H4	Memorability → psychological engagement	0.136	2.954	0.003 **	Supported
H5	Learnability → psychological engagement	0.186	3.629	0.000 ***	Supported
H6	Customizability → psychological engagement	0.068	1.550	0.121	Rejected
H7	Uncertainty avoidance → psychological engagement	−0.150	3.572	0.000 ***	Supported
H8	Psychological engagement → satisfaction	0.556	14.586	0.000 ***	Supported
H9	Psychological engagement → attachment	0.560	14.262	0.000 ***	Supported
H10	Satisfaction → stickiness	0.324	6.767	0.000 ***	Supported
H11	Attachment → stickiness	0.476	9.784	0.000 ***	Supported

Notes. Significance level: *** *p* < 0.001, ** *p* < 0.01.

**Table 6:** Mediation effect results.

Relationship	Total Effects	Specific Indirect Effects			
	β (t-Value)	Relationship	β (t-Value)	2.5%	97.5%
VA → ST	0.055 (2.649 **)	VA → PEN → SAT → ST	0.022 (2.428 *)	0.006	0.043
		VA → PEN → ATT → ST	0.033 (2.523 *)	0.010	0.061
PT → ST	0.052 (2.562 *)	PT → PEN → SAT → ST	0.021 (2.367 *)	0.005	0.040
		PT → PEN → ATT → ST	0.031 (2.444 *)	0.007	0.058
COM → ST	0.072 (3.269 **)	COM → PEN → SAT → ST	0.029 (2.935 **)	0.012	0.051
		COM → PEN → ATT → ST	0.043 (3.011 **)	0.018	0.074
MEM → ST	0.061 (2.941 **)	MEM → PEN → SAT → ST	0.025 (2.679 **)	0.008	0.044
		MEM → PEN → ATT → ST	0.036 (2.772 **)	0.012	0.063
LRN → ST	0.083 (3.373 **)	LRN → PEN → SAT → ST	0.034 (2.995 **)	0.014	0.058
		LRN → PEN → ATT → ST	0.050 (3.111 **)	0.021	0.084
CTM → ST	0.031 (1.523)	CTM → PEN → SAT → ST	0.012 (1.450)	−0.003	0.031
		CTM → PEN → ATT → ST	0.018 (1.502)	−0.005	0.043
UA → ST	−0.067 (3.424 **)	UA → PEN → SAT → ST	−0.027 (2.007 **)	−0.047	−0.011
		UA → PEN → ATT → ST	−0.040 (3.180 **)	−0.067	−0.017

Notes. Significance level: ** *p* < 0.01, and * *p* < 0.05.

## Data Availability

The data used to support the findings of this study are available from the corresponding author or first author upon request.

## Appendix A

**Table A1:** Definition of usability concepts.

Concept	Definition	Source
Completeness	The system can assist users in successfully completing tasks. This is usually measured objectively by system log files for the completion rate.	Brown et al. (2013, p. 1083)
Memorability	Users can easily remember how to perform tasks through the system.	
Learnability	Users are able to easily learn how to operate the system.	
Customizability	The system provides more than one way to accomplish tasks, which allows users to operate the system as preferred.	

## Appendix B

**Table A2:** Scale items per construct.

Construct	Measurement Items	Literature
Visual attractiveness (VA)	VA1: The user interface design of mobile health apps looks clean.	Fang et al. (2017)
	VA2: The user interface design of mobile health apps is sophisticated.	
	VA3: The user interface design of mobile health apps is fascinating.	
	VA4: The user interface design of mobile health apps is aesthetically pleasing.	
	VA5: The user interface design of mobile health apps is visually appealing.	
	VA6: The interface layout of mobile health apps is attractive.	
Prototypicality (PT)	PT1: This user interface looks very typical.	Miniukovich and Figl (2023); S. Lee et al. (2011)
	PT2: This user interface looks very exemplary.	
	PT3: Compared to other existing user interfaces, it meets my general expectations for a mobile health app user interface.	
Completeness (COM)	COM1: Provides me with complete information related to task management.	Tandon et al. (2024)
	COM2: Adequate user support helps me to complete my tasks easily.	
	COM3: Timely notifications provided by mHealth app UI update me about my health status.	
	COM4: Comprehensive notifications provided by mHealth app UI update me about my health status.	
Memorability (MEM)	MEM1: I can easily remember how to perform tasks using this UI.	Tandon et al. (2024)
	MEM2: The messages provided by this UI are easy to memorize.	
	MEM3: It can help me recall important health information whenever I go to the doctor, such as tasks or details, that I might otherwise forget.	
Learnability (LRN)	LRN1: I can easily learn how to use this user interface.	Tandon et al. (2024)
	LRN2: The mobile health user interface is easy to operate.	
	LRN3: I am able to manage my health check-ups using this UI.	
	LRN4: It is easy to become proficient in using this user interface and its features.	
Customizability (CTM)	CTM1: The user interface offers multiple methods to perform tasks.	Tandon et al. (2024)
	CTM2: I can always log on and use the app by using multiple tabs.	
	CTM3: It allows me to generate reports about my health issues through multiple means.	
	CTM4: Navigational structure is simple, and related information is placed together.	
Uncertainty avoidance (UA)	UA1: When starting a new job, I fear doing it.	Yoon (2009)
	UA2: I fear uncertainty about the future.	
	UA3: I fear ambiguous situations and unfamiliar adventures.	
	UA4: It is risky to do something that has never been done before.	
Psychological engagement (PEN)	PEN1: I feel strong and vigorous when I use mobile health apps.	Elsotouhy et al. (2024)
	PEN2: I am enthusiastic about using mobile health apps.	
	PEN3: Using mobile health apps is absorbing and immersive.	
Satisfaction (SAT)	STA1: I feel satisfied with using mobile health apps.	Elsotouhy et al. (2024)
	STA2: I feel content with using mobile health apps.	
	STA3: I feel pleased with using mobile health apps.	
	STA4: I am delighted with my overall experience of using mobile health apps.	
Attachment (ATT)	ATT1: I have a personal bond with mobile health apps.	Pedeliento et al. (2016)
	ATT2: Mobile health apps have a special role in my life.	
	ATT3: Mobile health apps are very dear to me.	
	ATT4: Mobile health apps mean a lot to me.	
	ATT5: I am very attached to mobile health apps.	
	ATT6: I feel emotionally connected to mobile health apps.	
Stickiness (ST)	SI1: I would stay for a long time while browsing mobile health apps.	M. Zhang et al. (2017)
	SI2: I intend to prolong my stays on mobile health apps.	
	SI3: I would visit this app frequently.	
